# Tipula (Vestiplex) crane flies (Diptera, Tipulidae) of Korea

**DOI:** 10.3897/zookeys.1061.49999

**Published:** 2021-09-30

**Authors:** Pavel Starkevich, Sigitas Podėnas, Virginija Podėnienė, Sun-Jae Park, A-Young Kim

**Affiliations:** 1 Nature Research Centre, Akademijos str. 2, LT-08412 Vilnius, Lithuania; 2 Life Sciences Centre of Vilnius University, Sauletekio str. 7, LT-10257 Vilnius, Lithuania; 3 Animal Resources Division, National Institute of Biological Resources, Incheon 22689, South Korea

**Keywords:** distribution, hypopygium, larva, new record, North Korea, ovipositor, South Korea, taxonomy

## Abstract

The Korean species of Tipula (Vestiplex) Bezzi, 1924 crane flies are taxonomically revised. Five species are recognized. Tipula (V.) coquillettiana Alexander, 1924, T. (V.) kuwayamai Alexander, 1921, T. (V.) tchukchi Alexander, 1934, and T. (V.) verecunda Alexander, 1924 are newly recorded from the Korean Peninsula, and T. (V.) serricauda Alexander, 1914 was previously recorded. The larva of T. (V.) serricauda is described and illustrated, and the larvae of the subgenus T. (*Vestiplex)* are divided into four groups based on spiracular lobe morphology. An identification key, redescriptions, and illustrations of Korean T. (Vestiplex) adults and grouping of known larvae are presented.

## Introduction

*Tipula* Linnaeus, 1758 is the largest genus in the family Tipulidae with a worldwide distribution, and it is divided into 41 subgenera. The subgenus T. (Vestiplex) Bezzi, 1924 is a terrestrial group represented by 177 species and subspecies recorded from Holarctic and Oriental regions (Oosterbroek, 2019). The highest diversity of this group is documented in the Eastern Palaearctic (76 species) and Oriental (74 species) regions (Oosterbroek 2019).

The first T. (Vestiplex) crane flies from the Korean Peninsula were collected by A.M. Yankovsky in 1938–1940. He lived and worked in the northern part of Korea. Only one species, T. (V.) serricauda Alexander, 1914, had been recorded from the Korean Peninsula (Starkevich et al. 2015).

The aim of this study was to document, redescribe, illustrate, and prepare keys for all Korean T. (Vestiplex) species.

## Material and methods

The specimen material examined in this paper (Table 1) was obtained from: the United States National Museum (USNM), Smithsonian Institution, Washington, DC, USA; the Snow Entomological Museum, University of Kansas (SEM), Lawrence, KS, USA; the National Institute of Biological Resources (NIBR), Incheon, South Korea and the Korea University (KU), Seoul, South Korea; the Academy of Natural Sciences of Drexel University (ANSP), Philadelphia, PA, USA; Zoological Museum of the Zoological Institute of the Russian Academy of Sciences, St. Petersburg, Russia (ZIN), and the Nature Research Centre (NRC), Vilnius, Lithuania.

**Table 1.** Collecting sites in Korea.

Locality	Year	Method	Collector	Museum	N* / E*
North Korea, Ompo (now called Onbo, Hamgyeongbuk-do,Gyeongsung-gun)	1937, 1938	Net	A.M. Yankovsky	USNM	41°30'48.9"N, 129°34'41.2"E
North Korea, Seren Mts.(Hamgyeongbuk-do, Gyeongsung-gun)	1938	Net	A.M. Yankovsky	USNM	41°41'14.3"N, 129°18'33.1"E
North Korea, Kankyo Nando, Puksu Pyaksan (now, Yanggang-do, Pungseo-gun, Mt Buksubaeksan)	1939	Net	A.M. Yankovsky	USNM	40°41'59.5"N, 127°42'57.6"E
North Korea, Chonsani (Yanggang-do, Daehongdan-gun)	1940	Net	A.M. Yankovsky	USNM	41°59'37.0"N, 128°45'09.0"E
Suoth Korea, # 8, Central National Forest, 18 miles NE of Seul	1954	Net	G.W. Byers	SEM	37°45'16.0"N, 127°09'57.4"E
South Korea, #14, Oho-ri, east coast	1954	Net	G.W. Byers	SEM	38°20'00.0"N, 128°30'00.0"E
South Korea, Gyeongi-do, Pocheon-si, Soheul-eup, Gwangneung Forest	1961	Net	Gyeong-suk Jeon	KU	37°45'02.8"N, 127°09'41.7"E
South Korea, Geongsanbuk-do, Chilgok-gun, Jicheon-myeon, Mt Hwanghaksan,	1978	Net	Seon-hui Lee	KU	36°02'06.5"N, 128°29'54.3"E
South Korea, Chungcheonnam-do, Danyang-gun, Danyang-eup, Mt Sobaeksan	1981	Net	K-S Lee	KU	36°57'06.9"N, 128°26'45.6"E
South Korea, Gyeonggi-do, Namyangju-si, Hwado-eup, Mt Cheonmasan	1984	Net	Yeong-cheol Heo	KU	37°40'50.4"N, 127°16'21.9"E
South Korea, Gyeonggi-do, Seongnam-si, Sangjeok-dong, Mt Cheongyesan	1984	Net	In-suk Hyeon	KU	37°24'51.4"N, 127°02'29.2"E
South Korea, Jeollanam-do, Suncheon-si, Songgwang-myeon, Mt Jogyesan	1988	Net	Dokgo	KU	35°00'09.0"N, 127°18'49.3"E
South Korea, Hadong-gun, Okjong-myeon, Wolhoeng-ri	1990	Net	M.J. Gang	NIBR	35°12'08.9"N, 127°50'56.1"E
South Korea, Chungcheongnam-do, Gongju-si, Gyeryong-myeon	1997	Net	Yeong Lee, Minjeong Kim	KU	36°22'11.3"N, 127°09'31.3"E
South Korea, Hadong-gun, Geumseong-myeon, Gadoek-ri, Hwaryeokbonbu	1998, 2000	Net	J.S. Jeon, J.S. Park	NIBR	34°57'23.2"N, 127°49'42.7"E
South Korea, Goseong-gun, Sangri-myeon, Osan-ri, Mt Odu	1999	Net	G.H. Gang, J.S. Jeon, J.S .Park, S.Y. Lee	NIBR	35°00'18.6"N, 128°11'12.4"E
South Korea, Geochang-gun, Gajo-myeon, Suwol-ri, Mt Bigye, Gogyeonsa	2000	Net	J.S. Choi, S.B. Jeong, S.H. Baek	NIBR	35°43'47.8"N, 128°02'16.1"E
South Korea, Gwangyang-si, Junggun-dong, Mt Gaya, Hanseokgwangwangnongwon	2000, 2001	Net	J.H. Son, J.S. Park, K.L. Han	NIBR	34°57'52.7"N, 127°41'03.8"E
South Korea, Ulju-gun, Sangbuk-myeon, Doekhyeon-ri, Mt Gaji, Helkijang	2001	Net	Y.S. Kim	NIBR	35°37'43.8"N, 129°00'58.6"E
South Korea, Gwangyang-si, Junggun-dong, Mt Gaya, Gunjangijae	2003	Net	T.H. An.	NIBR	34°58'17.8"N, 127°41'18.1"E
South Korea, Jirisan Hamyang, Songjeon-li Munsu-sa (Starkevich et. all 2015)	2005	Net	Tripotin	CMNH	35°24'44.4"N, 127°43'49.2"E
South Korea, Changpyeong-ri, Bongsung-myeon, Bonghwa-gun, Gyeongsangbuk-do	2014	Net	H.-W. Byun	NIBR	36°55'07.7"N, 128°48'39.4"E
South Korea, Jeollanam-do, Gurye-gun, Toji-myeon, Naeseo-ri, Jirisan National Park, Piagol valley	2014, 2016	Light	S. Podenas	NIBR	35°15'57.2"N, 127°34'51.5"E
South Korea, Jeollanam-do, Gurye-gun, Toji-myeon, Naeseo-ri, Jirisan National Park, Piagol valley	2015	Hand*	V. Podienene	NIBR	35°16'28.1"N, 127°33'49.6"E
South Korea, Jeollanam-do, Gurye-gun, Toji-myeon, Naeseo-ri, Jirisan National Park, Piagol valley	2016	Light	S. Podenas	NIBR	35°15'57.1"N, 127°34'51.2"E
South Korea, Jeollanam-do, Gurye-gun, Toji-myeon, Naeseo-ri, Jirisan National Park, Piagol valley	2016	Net	S. Podenas	NIBR	35°16'18.4"N, 127°34'17.3"E
South Korea, Jeollanam-do, Gurye-gun, Toji-myeon, Naeseo-ri, Jirisan National Park, Piagol valley	2016	Net	S. Podenas	NIBR	35°16'24.0"N, 127°34'09.3"E
South Korea, Jeju-do, Seogwipo, Sanghyo-dong	2017	Light	S. Podenas	NIBR	33°18'30.9"N, 126°33'34.0"E
South Korea, Jeju-do, Cheju, Jochon-eup, Seonheul-ri	2017	Light	S. Podenas	NIBR	33°30'35.8"N, 126°42'55.5"E
South Korea, Jeju-do, Jeju-si, Yonggang-dong	2017	Light	S. Podenas, V. Podeniene	NIBR	33°25'49.6"N, 126°35'50.5"E
South Korea, Gyeonggi-do, Gunpo-si, Suri-dong	2017	Light	S. Podenas	NIBR	37°21'02.1"N, 126°54'56.1"E
South Korea, Jeju-do, Seogwipo, Saekdal-dong	2019	Net	S. Podenas, H.-Y. Seo	NIBR	33°21'27.4"N, 126°27'51.2"E
South Korea, Jeju-do, Seogwipo, Saekdal-dong	2019	Net	S. Podenas	NIBR	33°21'37.6"N, 126°27'45.9"E

* Collecting site of larva.

Adult crane flies were collected by insect net and at lights. Some specimens were preserved dry in envelopes in the field and later mounted in the laboratory on their side on a paper point with legs generally surrounding the insect pin. The specimens are pinned except when noted otherwise.

Adult specimens were studied with a Nikon SMZ800 stereomicroscope. Photographs were taken with an INFINITY-1 camera mounted on a Nikon Eclipse 200 stereomicroscope and with a Canon EOS 80D camera mounted on an Olympus SZX10 dissecting microscope. Genitalia were studied after heating them in 10 percent NaOH solution for 5–10 minutes and then preserved in microvials filled with glycerol attached to the pin. All redescriptions and illustrations are based only on Korean material, except when otherwise mentioned.

Two identical last instar larvae were collected by hand and one of them was left for rearing. A female of T. (V.) serricauda emerged after 36 days. The larva is preserved in 70% ethanol though the head capsule was slide-mounted in glycerin jelly with corresponding label. The larva was studied with an Olympus SZX10 dissecting microscope with photographs taken with a Canon EOS 80D digital camera fitted with a Canon MP-E 65 mm macro lens.

Collecting localities with approximate coordinates are summarized in Table 1, and this was used to generate the geographical distribution maps (Figs 86–90). The identification key is based on morphological characters primarily observed in Korean specimens, but in cases when females are unknown from Korea, characters were observed from other specimens collected in other Asian countries.

Descriptive terminology of adults generally follows that of Cumming and Wood (2017). The term appendage of ninth sternite is adopted from Mannheims (1963), the terms ventral lobe and dorsal lobe of appendage of ninth sternite are adopted from Gelhaus (2005), the term gonocoxal fragment (= sclerites *sp1* and *sp2* (Neumann, 1958), = genital bridge (Dobrotworsky 1968)) for inner structure covered by ninth tergite is adopted from Brodo (2017). Descriptive terminology of larva generally follows that of Gelhaus (1986) and Neugart et al. (2009).

The overall world distribution of species is given according to Oosterbroek (2019).

## Taxonomy

### Tipula (Vestiplex)

Taxon classificationAnimaliaDipteraTipulidae

Bezzi, 1924

5821511E-1446-5241-A89E-36DD908EA58B

Tipula (Vestiplex) Bezzi 1924: 230; Edwards 1931: 79; Alexander 1934: 396; 1935: 117; 1965: 355; Mannheims 1953: 116; Savchenko 1964: 132.

#### Type species.

*Tipulacisalpina* Riedel, 1913.

*Vestiplex* was first proposed by Bezzi (1924) as a subgenus of the genus *Tipula* for the type species *T.cisalpina* Riedel, 1913, which was recorded from the Western Palaearctic (Italy and Switzerland). No fossil species of T. (Vestiplex) are described so far and only Matthews and Telka (1997) mentioned ovipositors of possibly T. (Vestiplex) females from Cape Deceit Formation in Western Alaska (1.8 MY old).

The world fauna of the subgenus T. (Vestiplex) includes 177 recent species and subspecies, which are distributed throughout the Nearctic, Palaearctic, and Oriental regions (Oosterbroek 2019). The majority of the species are associated with mountain systems (Pyrenees, Alps, Caucasus, and Himalayas) where adults are commonly found at altitudes ranging from 700 to 2500 m and rarely up to 4500 m (Savchenko 1960).

Females belonging to subgenus T. (Vestiplex) are characterized by the ovipositor having powerful cerci, which are heavily sclerotized, and serrated along outer margin (but smooth in several Asiatic species), and small to rudimentary hypovalva (Alexander 1935, 1965; Alexander and Byers 1981). The male genitalia are extremely polymorphic (Savchenko 1964), typically with the ninth tergite forming a shallowly concave and sclerotized saucer, with other species having their ninth tergite completely divided longitudinally by a pale membrane (Alexander 1935; Alexander and Byers 1981).

Just seven species have described larvae. Two are North American species, T. (V.) arctica Curtis, 1835 and T. (V.) platymera Walker, 1856 (Alexander 1920a, Gelhaus 1986), and five are European species, T. (V.) excisa excisa Schummel, 1833, T. (V.) hortorum Linnaeus, 1758, T. (V.) nubeculosa Meigen, 1804, T. (V.) semivittata Savchenko, 1960, and T. (V.) scripta Meigen, 1830 (Chiswell 1956; Theowald 1965; Savchenko 1986; Podeniene 2003; Lantsov 2003).

The immature stages develop in terrestrial habitats such as the uppermost layer of soil under leaf or needle litter, or under mosses (Chiswell 1956; Rogers 1942; Theowald 1967; Teale and Gelhaus 1984; Lantsov 2003; Podeniene 2003). Larvae of T. (Vestiplex) are easily recognized because of a brown band separating the anus from the anal papillae. The lobes surrounding spiracular field are subconical with the lateral lobe situated dorsolaterally. The sclerotization of the dorsal lobe varies depending on species, with some species bearing a sclerite only on the basal part of the posterior surface, while in other species the entire anterior and posterior surfaces are sclerotized. In this case the apex of the dorsal lobe is sclerotized, pointed, and directed anteriorly. The lateral lobe may possess a narrow and vertical sclerite, but it may be entirely absent in some species. The ventral lobe is the smallest and trianglar. It varies from extensively sclerotized to possessing only a small sclerite. Larvae have two pairs of short, blunt anal papillae. The length and macrosetal arrangement is consistent on the dorsum and tergum among all known species. Short microscopic hairs are arranged in transverse rows and cover most of the abdomen.

##### List of Korean Tipula (Vestiplex) crane flies

Tipula (Vestiplex) coquillettiana Alexander, 1924

Tipula (Vestiplex) kuwayamai Alexander, 1921

Tipula (Vestiplex) serricauda Alexander, 1914

Tipula (Vestiplex) tchukchi Alexander, 1934

Tipula (Vestiplex) verecunda Alexander, 1924

### Key to Korean Tipula (Vestiplex) crane flies

**Table:** 

**Males**		
1	Gonocoxite armed with a powerful black spine or bifurcate horn (Figs 3, 32)	**2**
–	Gonocoxite simple, unarmed (Figs 16, 71)	**3**
2	Flagellum bicolored with inconspicuous basal enlargement. Gonocoxite with a strong black spine (Fig. 32). Ninth tergite divided by pale midline (Fig. 31)	**Tipula (Vestiplex) serricauda**
–	Flagellum dark brown with weak basal enlargement. Gonocoxite horn-shaped with bifurcate margin (Fig. 3). Ninth tergite forming narrow sclerotised saucer (Fig. 2)	**Tipula (Vestiplex) coquillettiana**
3	Eighth sternite with long setae (Figs 67, 70) . Ninth sternite ventrally with median tubercle (Fig. 67). Appendage of ninth sternite present, finger-shaped (Figs 74, 75). Ninth tergite divided by pale midline (Fig. 68)	**Tipula (Vestiplex) verecunda**
–	Eighth sternite without long setae (Fig. 16). Ninth sternite without ventral tubercle (Fig. 16). Appendage of ninth sternite absent. Ninth tergite forming a sclerotised saucer (Figs 17, 56)	**4**
4	Size relatively small (body length 16.8 mm, wing length 17.1 mm). Wing pattern indistinct, only weak darkening along vein CuA. Abdomen with median stripe	**Tipula (Vestiplex) tchukchi**
–	Size large (body length 17.8–19.7 mm, wing length 19.8–22.9 mm). Wing distinctly marbled. Abdomen without median stripe	**Tipula (Vestiplex) kuwayamai**
**Females**		
1	Wing well developed, extends beyond middle of abdomen	**2**
–	Wing reduced, not reaching middle of abdomen	**Tipula (Vestiplex) coquillettiana**
2	Wing pattern indistinct, only weak darkening along vein CuA. Cercus apically with incision in addition to serrated border (Fig. 65)	**Tipula (Vestiplex) tchukchi**
–	Wing pattern distinctly marbled. Cercus without apical incision (Figs 11, 27, 38, 80)	3
3	Antennal flagellum brownish-black. Cercus with smooth ventral margin (Fig. 80). Hypovalva long, blade-shaped (Figs 81, 82)	**Tipula (Vestiplex) verecunda**
–	Antennal flagellum yellow or bicolored. Cercus with serrate ventral margin (Fig. 27, 38). Hypovalva short, filamentous or plate-shaped (Figs 28, 39)	**4**
4	Large species with wing length 24.5–26.1 mm. Antennal flagellum bicolored. Hypovalva filamentous (Fig. 28)	**Tipula (Vestiplex) kuwayamai**
–	Smaller species with wing length16.4–17.1 mm. Antennal flagellum varies from yellow to bicolored. Hypovalva in the shape of a dark brown plate (Fig. 39)	**Tipula (Vestiplex) serricauda**

### Tipula (Vestiplex) coquillettiana

Taxon classificationAnimaliaDipteraTipulidae

Alexander, 1924

4C9EB13F-DAC9-5103-BB39-6A668828CD2F

Figs 1–10 , 11–15 , 86

Tipula coquillettiana Alexander 1924: 605; 1925: 91.
Tipula (Vestiplex) coquillettiana : Alexander 1934: 405; 1935: 118; Savchenko 1960: 172; 1964: 180; Oosterbroek and Theowald 1992: 154.

#### Type material examined.

Holotype, male, **RUSSIA**, Odasam [Southern Sakhalin], 5 August 1922, Esaki; paratype, male (USNM).

#### Other examined material

(Fig. 86). **NORTH KOREA**, 1 male, Seren Mts, alt. 3000 ft, 25 June 1938, Yankovsky (USNM); 2 males, alt. 3000 ft, 26 June 1938, Yankovsky (USNM); 2 males, alt. 3000–4000 ft, 29–30 June 1938, Yankovsky (USNM); 1 male, alt. 3500–5500 ft, 29–30 June 1938, Yankovsky (USNM); 3 males, alt. 4000–5000 ft, 29–30 June 1938, Yankovsky (USNM); 1 male, alt. 5000 ft, 29–30 June 1938, Yankovsky (USNM); 2 females, alt. 4000 ft, 30 June 1938, Yankovsky (USNM); 2 males, alt. 3500 ft, 5 July 1938, Yankovsky (USNM); 3 males, alt. 4500 ft, 5–6 July 1938, Yankovsky (USNM); 1 male, alt. 4000 ft, 10–11 July 1938, Yankovsky (USNM); 1 male, alt. 5500 ft, 18 July 1938, Yankovsky (USNM); 1 male, Kankyo Nando Puksu Pyaksan, alt. 6000 ft, 23 June 1939, Yankovsky (USNM); 1 male, alt. 6000 ft, 31 July 1939, Yankovsky (USNM); 1 male, alt. 5000 ft, 2 August 1939, Yankovsky (USNM); 2 males, Chonsani, alt. 4000 ft, 2 June 1940, Yankovsky (USNM); 1 male, alt. 3000 ft, 8 June 1940, Yankovsky (USNM); 4 males, alt. 4500 ft, 20 June 1940, Yankovsky (USNM); 1 male, alt. 4000 ft, 23 June 1940, Yankovsky (USNM).

#### Diagnosis.

Tipula (V.) coquillettiana can be easily recognized by the ninth tergite, which forms a narrow, saucer-shaped plate, and the horn-shaped gonocoxite. The tip of the gonocoxite is bifurcate. The female of this species has a gray, elongated abdomen and greatly reduced wing. The cercus is straight with its tip narrowed and the ventral margin and apical part of the dorsal margin distinctly serrated. The hypovalva is in the shape of an elongated filament.

***Male.***

Body length 17.1–21.3 mm, wing length 18.9–22.8 mm. General body coloration brownish yellow.

*Head*. Vertex and occiput gray with dark median line. Rostrum yellowish, thinly dusted with gray dorsally. Nasus distinct. Antenna 13-segmented, if bent backward extending beyond wing base. Scape and pedicel yellow; first flagellar segment basally yellow, distally brownish black; succeeding flagellar segments brownish black. Each flagellomere, except first, with basal enlargement and small incision. Apical flagellomere small, reduced. Verticils approximately as long as corresponding segments. Palpus with first segment yellowish, second brownish yellow, and other segment brownish black.

*Thorax*. Pronotum yellowish, thinly dusted with gray. Prescutum and presutural scutum gray; stripes bluish gray bordered by brown. Postsutural scutum, scutellum, and postnotum gray with bluish shade; all sclerites with dark, median line. Pleura brownish, dusted with gray. Coxa gray; trochanter yellowish; femur brownish yellow with tip broadly darkened; tibia brownish yellow; tarsal segments dark brown. Tarsal claw without tooth. Wing patterned with brown. Halter brownish yellow with brown knob.

*Abdomen*. Yellow. First abdominal segment dusted with gray. Abdominal segments 2 and 3 yellow, 4 and 5 brownish-yellow, and remaining segments dark brown. Lateral margin of tergites pale yellow. Dorsal median stripe pale, broadly interrupted.

**Figures 1–10.**
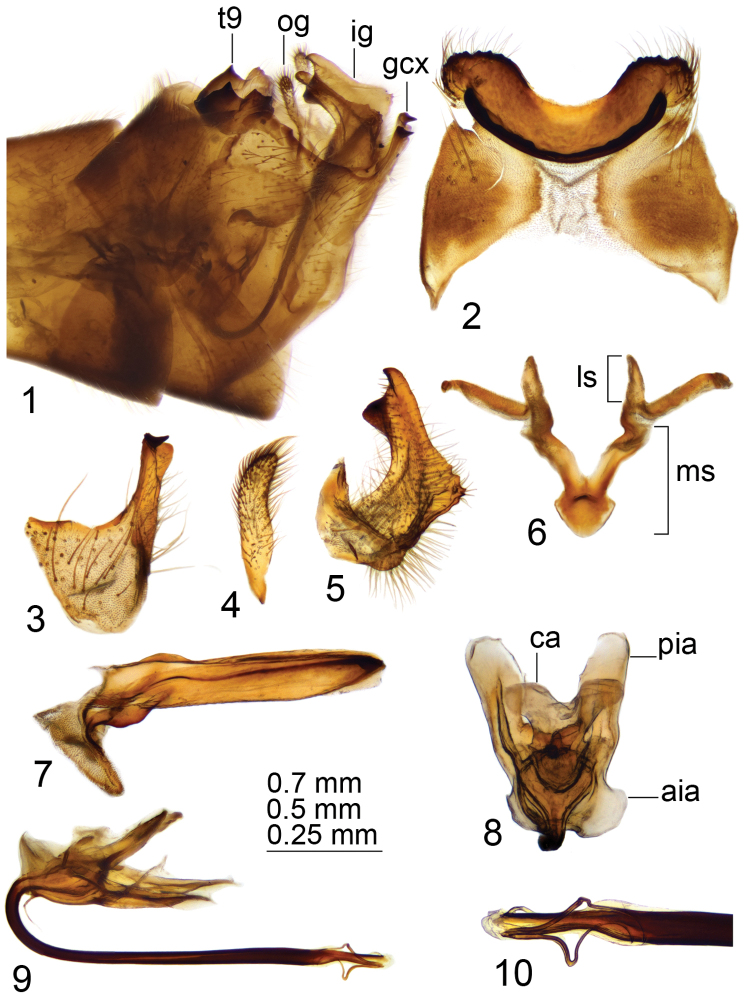
Male terminalia of T. (Vestiplex) coquillettiana**1** hypopygium, lateral view **2** ninth tergite, dorsal view **3** left gonocoxite, lateral view **4** left outer gonostylus **5** left inner gonostylus, lateral view **6** gonocoxal fragment, dorsal view **7** adminiculum, lateral view **8** semen pump, dorsal view **9** semen pump and intromittent organ, lateral view **10** distal part of intromittent organ, lateral view. Abbreviations: aia, anterior immovable apodeme; ca, compressor apodeme; gcx, gonocoxite; ig, inner gonostylus; ls, lateral sclerite of gonocoxal fragment; ms, medial sclerite of gonocoxal fragment; og, outer gonostylus; pia, posterior immovable apodeme; t9, ninth tergite. Scale bars: 0.7 mm (**1**); 0.5 mm (**2–9**); 0.25 mm (**10**).

*Hypopygium*. Brown (Fig. 1). Ninth tergite distally forming a narrow saucer-shaped plate (Figs 1, 2). Tergal saucer posteriorly with broad median notch; lateral comer with serrated and blackened margin, provided with setae. Anterior margin elevated into narrow, blackened rim reaching lateral part of tergal saucer and terminating into acute projection. Gonocoxite apically extended, with tip split into blackened beak pointed caudad and rounded projection (Figs 1, 3). Outer gonostylus slightly curved, finger-shaped with tip narrowed (Fig. 4). Inner gonostylus in the shape of a curved plate; beak blackened, triangular; dorsal margin claw-shaped; middorsal edge extended and serrated (Fig. 5). Gonocoxal fragment with flattened medial sclerites, fused into broad, nearly triangular base (Fig. 6). Lateral sclerite flattened and bilobed. Adminiculum boat-shaped, with tip and dorsal edge apically blackened (Fig. 7). Semen pump with flattened central vesicle (Figs 8, 9). Compressor apodeme with round median incision, forming a 30° angle with posterior immovable apodeme. Posterior immovable apodeme much longer than compressor apodeme, flattened, rounded apically. Anterior immovable apodeme flattened, rounded. Intromittent organ tube-shaped, about twice as long as semen pump, brownish-black. Distal part with preapical yellow membrane, apex with pale median incision, lateral parts split, filament-shaped (Fig. 10).

***Female*.** Body length 26.9–30.2 mm, wing length 4.7–5.7 mm. Generally similar to male, but with elongated and gray abdomen. Tergites and sternites with pale margins. Wing greatly reduced.

**Figures 11–15.**
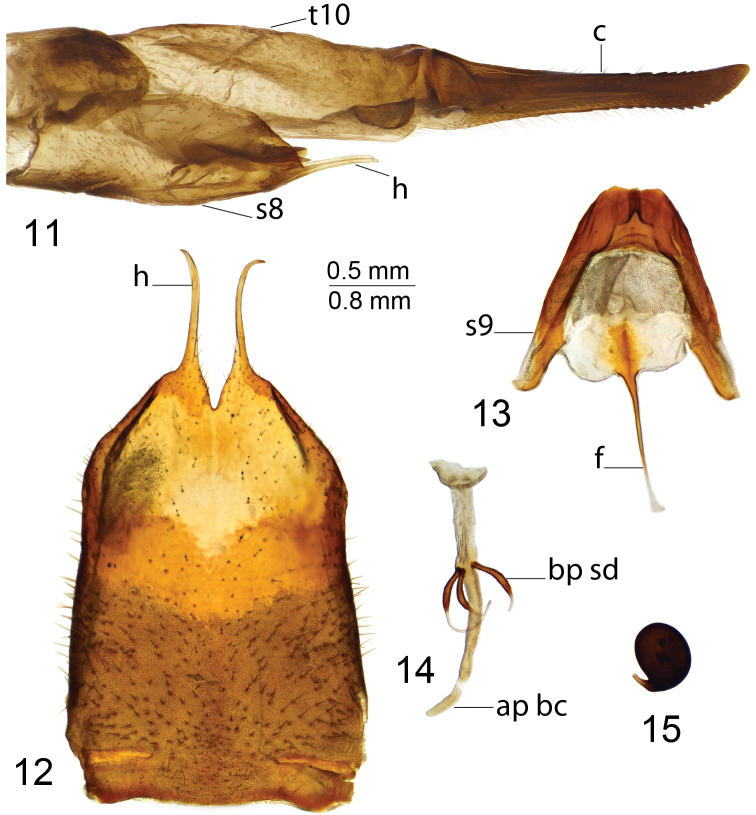
Female terminalia of T. (Vestiplex) coquillettiana**11** ovipositor, left lateral view **12** eighth sternite with hypovalvae, ventral view **13** ninth sternite with furca, dorsal view **14***bursacopulatrix*, dorsal view **15** spermatheca, lateral view. Abbreviations: ap bc, anterior part of *bursacopulatrix*; bp sd, basal part of spermathecal duct; c, cerci; f, furca; h, hypovalvae; s8, eighth sternite; s9, ninth sternite; t10, tenth tergite. Scale bars: 0.8 mm (**11**), 0.5 mm (**12–14**).

*Female terminalia*. Tenth tergite shining dark brown. Cercus brown, straight, as long as tenth tergite, with tip narrowed; ventral margin and apical part of dorsal margin distinctly serrated (Fig. 11). Hypovalva elongated and filamentous (Fig. 12). Median incision between hypovalvae deeper than posterior margin of eighth sternite. Lateral angle of eighth sternite sloping. Ninth sternite with lateral parts straight (Fig. 13). Furca anteriorly narrowed, shaped posteriorly as broad membranous plate (Fig. 13). *Bursacopulatrix* with spermathecal duct sclerotized at base, in shape of thickened, curved stick (Fig. 14). Spermatheca broadened at base, pear-shaped (Fig. 15).

#### Known distribution.

Russia, Kazakhstan, and Japan (Oosterbroek 2019). Recorded here for the first time from North Korea.

### Tipula (Vestiplex) kuwayamai

Taxon classificationAnimaliaDipteraTipulidae

Alexander, 1921

785171E0-B0BF-5C0A-B7BC-765FDB888DCA

Figs 16–26 , 27–29 , 87

Tipula kuwayamai Alexander 1921: 130; 1925: 93.
Tipula (Vestiplex) kuwayamai : Alexander 1934: 405; 1935: 118; Savchenko 1964: 179; Oosterbroek and Theowald 1992: 156.

#### Type material examined.

Holotype, male, **JAPAN**, Maruyama, Sapporo, 1 June 1919, Kuwayama (USNM).

#### Other examined material

(Fig. 87). **NORTH KOREA**, 4 males, Ompo, 23 May 1937, Yankovsky (USNM); 1 female, alt. 500 ft, 2 May 1938, Yankovsky (USNM); 1 male, alt. 400 ft, 10 May 1938, Yankovsky (USNM); 1 male, Chonsani, alt. 4900 ft, 2 June 1940, Yankovsky (USNM); 1 female, alt. 3500 ft, 13 June 1940, Yankovsky (USNM). **SOUTH KOREA**, 1 male, #8, Central National Forest, 18 miles NE of Seoul, 28 May 1954, G.W. Byers (SEM); 1 male, Gyeongi-do, Pocheon-si, Soheul-eup, Gwangneung Forest, 30 May 1961, Gyeong-suk Jeon (KU); 1 female, Chungcheonnam-do, Danyang-gun, Danyang-eup, Mt Sobaeksan, 6 June 1981, K-S Lee (KU); 1 female, Gyeonggi-do, Seongnam-si, Sangjeok-dong, Mt Cheongyesan, 4 May1984, In-suk Hyeon (KU); 1 female, Gyeonggi-do, Namyangju-si, Hwado-eup, Mt Cheonmasan, 20 May 1984, Yeong-cheol Heo (KU); 1 male, 3 females, Geochang-gun, Gajo-myeon, Suwol-ri, Mt Bigye, Gogyeonsa, 6–7 May 2000, S.B. Jeong, IN0000297019, IN0000297023, IN0000296935, IN0000297021 (NIBR); 1 female, S.H. Baek, IN0000296936 (NIBR); 1 female, J.S. Choi, IN0000297020 (NIBR); 1 female, Ulju-gun, Sangbuk-myeon, Doekhyeon-ri, Mt Gaji, Helkijang, 18–19 May 2001, Y.S. Kim, IN0000226477 (NIBR). **CHINA**, 1 male, Heilongjiang Province, Hsiaoling, 20 May 1938, leg. Weymarn (USNM); 6 males, 2 females, Heilongjiang Province, Maoershan, 8 June 1941, [collector not designated] (USNM); 1 female, 11 June 1941 (USNM); 1 male, 13 June 1941 (USNM); 1 female, 14 June 1941 (USNM); 1 female, 16 June 1941 (USNM); 1 male, Jilin Province, Yablonia Station [Yabuli], 26 May 1939, [collector not designated] (USNM).

#### Diagnosis.

Tipula (V.) kuwaymai can be recognized by the unarmed gonocoxite and by the ninth tergite forming a sclerotized, oval saucer which has an elevated edge anteriorly and is yellow posteriorly with the posterolateral angle blade-shaped. The wing is distinctly patterned with brown. The female has the cercus brownish yellow with the tip narrowed and upturned, and the ventral margin has distinct serration. The hypovalva is filamentous. The median incision between hypovalvae is slightly deeper than posterior margin of eighth sternite.

***Male*.** Body length 17.8–19.7 mm, wing length 19.8–22.9 mm. General body coloration brownish yellow.

*Head*. Vertex and occiput gray with brown median line. Rostrum dark brown, dorsally dusted with gray. Nasus small, almost lacking. Antenna 13-segmented, if bent backward extending beyond the wing base. Scape and pedicel reddish yellow, flagellum dark brown. Each flagellomere except first one with distinct basal enlargement and incision. Apical flagellomere small, reduced. Verticils longer than corresponding segments. Palpus dark brown.

*Thorax*. Pronotum gray with brown median line. Prescutum and presutural scutum gray. Median stripes anteriorly gray, posteriorly brown, bordered by darker brown, fused at base. Lateral stripes blackish gray, bordered by brown. Postsutural scutum blackish gray; each lobe with light brown spot bordered by brown. Scutellum and postnotum brownish, dusted with gray, each with brown median line. Pleura brownish, dusted with gray. Wing distinctly patterned with brown. Halter pale with brown knob. Coxa gray; trochanter yellow; femur brownish yellow with tip dark brown; tibia and tarsal segments brown. Tarsal claw with tooth.

*Abdomen*. Brownish yellow. First tergite laterally brown, dorsally yellow, tergites 2–5 yellow with pale and interrupted dorsal stripe. Tergites 6–9 brown, without median stripe. Lateral stripe distinct. First sternite yellow, sternites 2–6 yellow; remaining sternites dark brown.

**Figures 16–26.**
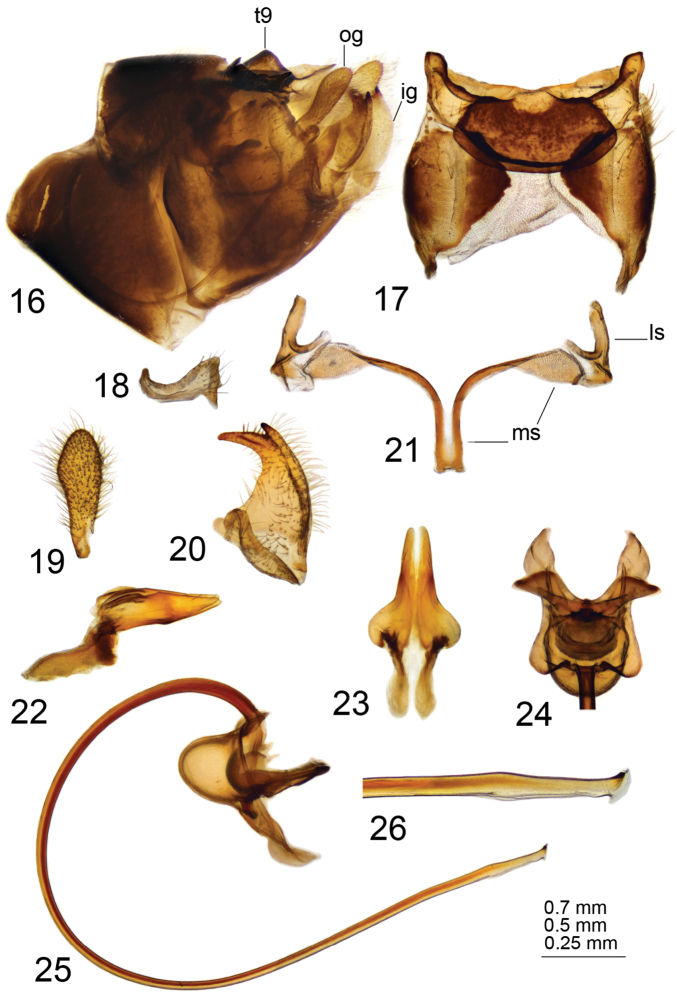
Male terminalia of T. (Vestiplex) kuwayamai**16** hypopygium, lateral view **17** ninth tergite, dorsal view **18** left gonocoxite, lateral view **19** left outer gonostylus **20** left inner gonostylus, lateral view **21** gonocoxal fragment, dorsal view **22** adminiculum, lateral view **23** adminiculum, ventral view **24** semen pump, dorsal view **25** semen pump and intromittent organ, lateral view **26** distal part of intromittent organ, lateral view. Abbreviations: ig, inner gonostylus; ls, lateral sclerite of gonocoxal fragment; ms, medial sclerite of gonocoxal fragment; og, outer gonostylus; t9, ninth tergite. Scale bars: 0.7 mm (**16**); 0.5 mm (**17–25**); 0.25 mm (**26**).

*Hypopygium*. Dark brown (Fig. 16). Ninth tergite forming concave sclerotized saucer (Fig. 17). Central part of tergal saucer brownish, in the shape of an oval, concave, transverse plate, anteriorly with elevated edge, laterally and posteriorly with blackened, medially interrupted rim. Posterior part of tergal saucer yellow, posterolateral angle blade-shaped, with blackened ridge connecting with central part of tergal saucer. Gonocoxite narrow, unarmed (Fig. 18). Outer gonostylus club-shaped (Fig. 19). Inner gonostylus in the shape of a curved plate, bidentate at apex, dorsally with acute tooth, beak claw-shaped (Fig. 20). Gonocoxal fragment with medial sclerites slender, fused at base; lateral parts apically flattened (Fig. 46). Lateral sclerite relatively small, nearly V-shaped. Adminiculum semi-open, nearly triangular in lateral view (Fig. 22), flattened and broadened at base in ventral view (Fig. 23). Semen pump with swollen central vesicle (Figs 24, 25). Compressor apodeme with broad and round median incision, forming a 75° angle with posterior immovable apodeme. Posterior immovable apodeme much longer than compressor apodeme, flattened, with acute tip. Anterior immovable apodeme narrow, rounded. Intromittent organ tube-shaped, about five times as long as semen pump, basally and medially brown but passing into brownish yellow towards apex. Distal part yellow, funnel-shaped (Fig. 26).

***Female*.** Body length 27.4–34.4 mm, wing length 24.5–26.1 mm. Generally similar to male. Antenna with four basal segments yellow; remaining flagellomeres bicolored.

**Figures 27–29.**
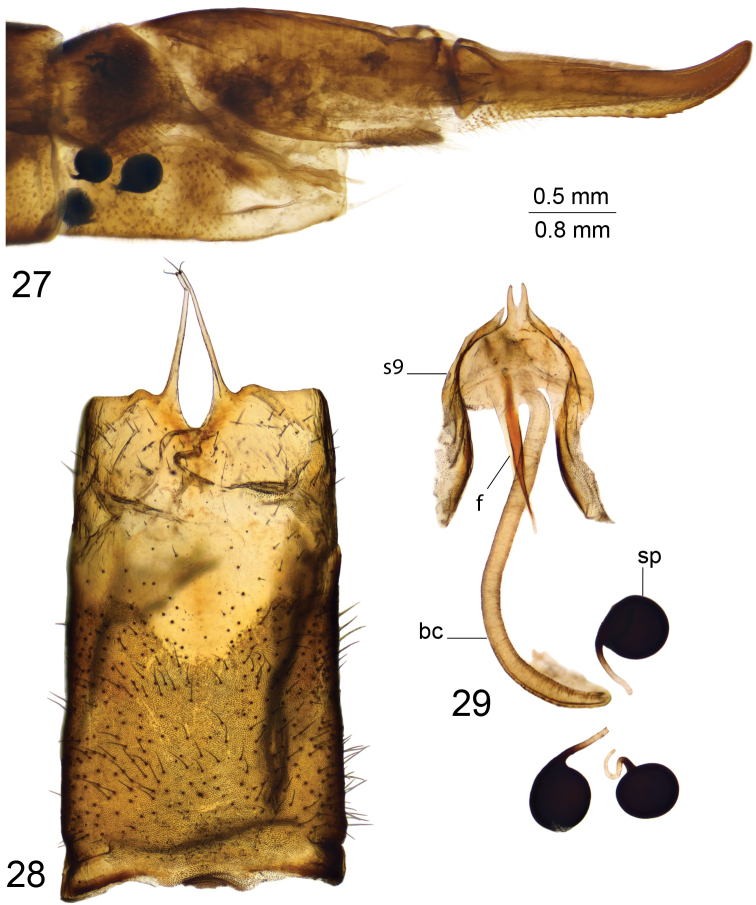
Female terminalia of T. (Vestiplex) kuwayamai**27** ovipositor, left lateral view **28** eighth sternite with hypovalvae, ventral view **29** ninth sternite with furca, *bursacopulatrix* and spermathecae, dorsal view. Abbreviations: bc, *bursacopulatrix*; f, furca; s9, ninth sternite; sp, spermatheca. Scale bars: 0.8 mm (**27**); 0.5 mm (**28–29**).

*Female terminalia*. Tenth tergite shining-brownish. Cercus brownish yellow, as long as tenth tergite, with tip narrowed and upturned, ventral margin with distinct serration (Fig. 27). Hypovalva filamentous, distally pale, with short trichia at tip (Fig. 28). Median incision between hypovalvae slightly deeper than posterior margin of eighth sternite. Lateral incision scarcely outlined, posterior margin with fine additional projection. Lateral angle of eighth sternite rectangular. Ninth sternite posteriorly with split tip (Fig. 29). Furca in the shape of narrow stripe (Fig. 29). Spermatheca spherical (Fig. 29).

#### Known distribution.

Russia, Japan China (Oosterbroek, 2019) and North and South Korea. Recorded here for the first time from the Korean Peninsula.

### Tipula (Vestiplex) serricauda

Taxon classificationAnimaliaDipteraTipulidae

Alexander, 1914

763EFE45-2ADC-547B-8678-C471770B7DFE

Figs 30–37 , 38–41 , 42–46 , 47–53 , 54–55 , 88

Tipula serricauda Alexander 1914: 237; 1920b: 18.
Tipula asio Alexander 1918: 68 (synonymy after Alexander 1953: 156).
Tipula (Vestiplex) asio : Alexander 1935: 118.
Tipula (Vestiplex) serricauda : Alexander 1935: 118; Oosterbroek and Theowald 1992: 159.

#### Type material examined.

Holotype, female, **JAPAN**, Tokyo, August 1912 (USNM).

#### Other examined material

(Fig. 88). **SOUTH KOREA**, 2 males, #14, Oho-ri, east coast, 10–50 ft, 128°30'E, 38°20'N, 11 June 1954, G.W. Byers (SEM); 1 female, Geongsanbuk-do, Chilgok-gun, Jicheon-myeon, Mt Hwanghaksan, 4 June 1978, Seon-hui Lee (KU); 1 male, 1 female, Jeollanam-do, Suncheon-si, Songgwang-myeon, Mt Jogyesan, 23 May 1988, Dokgo (KU); 1 male, Hadong-gun, Okjong-myeon, Wolhoeng-ri, 24 May 1990, M.J. Gang, IN0000296230 (NIBR); 1 male, Chungcheongnam-do, Gongju-si, Gyeryong-myeon, 5–7 June 1997, Yeong Lee (KU); 1 male, Minjeong Kim (KU); 1 male, Hadong-gun, Geumseong-myeon, Gadoek-ri, Hwaryeokbonbu, 19–20 September 1998, J.S. Jeon, IN0000298240 (NIBR); 3 males, 1 female, Goseong-gun, Sangri-myeon, Osan-ri, Mt Odu, 10–11 September 1999, J.S. Jeon, IN0000298933, IN0000298927, IN0000298197, IN0000297995 (NIBR); 3 females, J.S. Park, IN0000298194, IN00000298192, IN0000298188 (NIBR); 2 males, 1 female, S.Y. Lee, IN0000298932, IN0000298935, IN0000298193 (NIBR); 2 females, G.H.Gang, IN0000298196, IN0000298187(NIBR); 2 females, Hadong-gun, Geumseong-myeon, Gadoek-ri, Hwaryeokbonbu, 22–23 September 2000, J.S. Park, NIBR IN0000333964 (NIBR); 1 male, Gwangyang-si, Junggun-dong, Mt Gaya, Hanseokgwangwangnongwon, 22–23 September 2000, J.H. Son, IN0000333965; 2 females, K.L. Han, IN0000333962, IN0000333956 (NIBR); 1 male, Gwangyang-si, Junggun-dong, Mt Gaya, Hanseokgwangwangnongwon, 26–27 May 2001, J.S. Park, IN0000323569 (NIBR); 2 females, Gwangyang-si, Junggun-dong, Mt Gaya, Gunjangijae, 31 May–1 June 2003, T.H. An, IN0000298937, IN0000299044 (NIBR); 1 male, Changpyeong-ri, Bongsung-myeon, Bonghwa-gun, Gyeongsangbuk-do, 36°55.12'N, 128°48.65'E, 2014.05.05, H.-W. Byun (NIBR); 1 male, Jeollanam-do, Gurye-gun, Toji-myeon, Naeseo-ri, Jirisan National Park, Piagol valley, 35°15.95'N, 127°34.85'E, alt. 450 m, 24 August 2014, S. Podenas (NIBR); 1 female, 1 larva, Piagol valley, 35°16.47'N, 127°33.82'E, alt. 600 m, 1 May 2015, female emerged 5 June 2015, V. Podeniene (NIBR); 1 male, 2 females, Piagol valley, 35°15.95'N, 127°34.85'E, alt. 450 m, 2 June 2016, S. Podenas (NIBR); 1 male, 6 females, Piagol valley, 35°15.95'N, 127°34. 85'E, alt. 450 m, 3 June 2016, S. Podenas (NIBR); 16 males, 4 females, Piagol valley, 35°15.95'N, 127°34.85'E, alt. 450 m, 3 June 2016, S. Podenas (NIBR); 1 male, 2 females, Piagol valley, 35°16.31'N, 127°33.29'E, alt. 500 m, 3 June 2016, S. Podenas (NIBR); 2 males, 3 females, Piagol valley, 35°16.4'N, 127°34.15'E, alt. 550 m, 3 June 2016, S. Podenas (NIBR); 8 males (in alcohol), Piagol valley, 35°15.95'N, 127°34.85'E, alt. 450 m, 24 June 2016 S. Podenas, at light (NIBR); 4 males (in alcohol), 26 June 2016, S. Podenas, at light (NIBR); 3 males (in alcohol), 2 males, 2 females, Jeju-do, Jeju Island, Seogwipo, Sanghyo-dong, 33°18.51'N, 126°33.57'E, alt. 500 m, 22 May 2017, S. Podenas (NIBR); 2 males, Jeju-do, Jeju Island, Cheju, Jochon-eup, Seonheul-ri, 33°30.59'N, 126°42.92'E, alt. 1500 m, 23 May 2017, at light, S. Podenas (NIBR); 1 male, Jeju-do, Jeju Island, Jeju-si, Yonggang-dong, 33°25.82'N, 126°35.84'E, alt. 600 m, 24 May 2017, at light, S. Podenas, V. Podeniene (NIBR); 1 male, Gyeonggi-do, Gunpo-si, Suri-dong, 37°21.03'N, 126°54.93'E, alt. 140 m, 27 May 2017, S. Podenas, at light, (NIBR); 1 male, 1 female (in alcohol), Jeju-do, Jeju Island, Seogwipo, Saekdal-dong, 33°21.45'N, 126°27.85'E, alt. 1100 m, 19 June 2019, S. Podenas (NIBR); 1 male, 18 June 2019, H.-Y. Seo (NIBR). Also material listed by Starkevich et al. (2015).

#### Diagnosis.

Tipula (V.) serricauda can be recognized by the gonocoxite being armed with a black spine and the ninth tergite divided by pale membrane with ventral portion yellow and bearing a pair of blackened lobes. The body is yellowish, with short antenna reaching the pronotum if bent backward. Female can be recognized by the short, plate-shaped hypovalvae.

***Male*.** Body length 12.9–17.3 mm, wing length 15.6–20.2 mm. General body coloration yellowish.

*Head*. Yellowish dusted with gray, vertex and occiput yellowish, with dark brown median line. Rostrum yellowish with conspicuous nasus. Antenna 12-segmented, if bent backward reaching pronotum. Scape, pedicel, the first and second flagellar segments yellow; flagellar segments 3–10 darkened at base and yellow apically; remaining segments dark brown. Each flagellomere, except first one, with small, inconspicuous enlargement. Apical flagellomere very small, reduced, distinctly shorter than preceding segment. Verticils longer than corresponding segments. Palpus dark brown.

*Thorax*. Brownish yellow. Pronotum gray with brown median line. Prescutum and presutural scutum with four longitudinal, grayish-brown stripes bordered by darker brown. Intermediate pair brownish, fused anteriorly and posteriorly, separated in the first third. Lateral stripe grayish. Interspace between median and lateral stripes light brown. Postsutural scutum yellowish, dusted with light gray; scutal lobe with two yellowish spots. Scutellum yellowish; postnotum yellowish, lightly dusted with gray with brown median line. Pleura brownish yellow, lightly dusted with gray. Coxa yellowish; trochanter yellow; femur yellow, distally brown; tibia yellowish brown; tarsal segments dark brown; claw with tooth. Wing distinctly patterned with brown. Halter yellow, knob brown with distal part pale yellow.

*Abdomen*. Brownish yellow, tergites 1 and 2 with brown spots; tergites 3–5 with dorsal stripe which varies from pale to brown; tergites 6–9 with dorsal stripe distinct, dark brown. Lateral stripe distinct, dark brown. Posterior and lateral margins of tergites pale. First sternite yellowish, sternites 2–4 reddish yellow, remaining sternites passing into brown.

**Figures 30–37.**
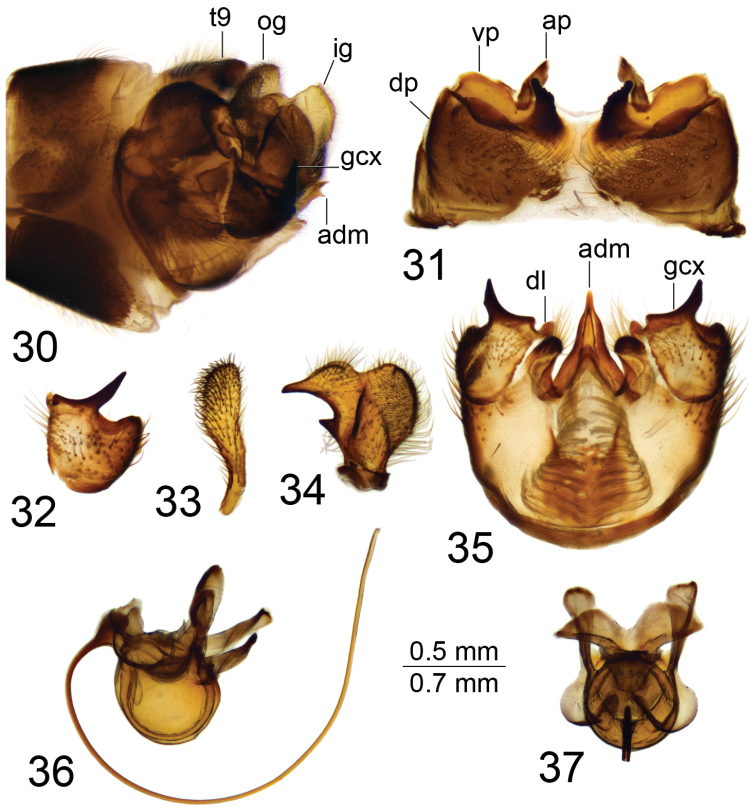
Male terminalia of T. (Vestiplex) serricauda**30** hypopygium, lateral view **31** ninth tergite, dorsal view **32** left gonocoxite, lateral view **33** left outer gonostylus **34** left inner gonostylus, lateral view **35** ninth sternite, ventral view (ninth tergite, outer and inner gonostyles removed) **36** semen pump and intromittent organ, lateral view **37** semen pump, dorsal view. Abbreviations: adm, adminiculum; ap, anal plate; dl, dorsal lobe of appendage of ninth sternite; dp, dorsal portion of ninth tergite; gcx, gonocoxite; ig, inner gonostylus; og, outer gonostylus; t9, ninth tergite; vp, ventral portion of ninth tergite. Scale bars: 0.7 mm (**30**); 0.5 mm (**31–37**).

*Hypopygium*. Brownish (Fig. 30). Ninth tergite divided by pale membrane; dorsal portion brown medially, provided with long setae; ventral portion yellow with a pair of blackened, microscopically roughened lobes on either side of midline (Fig. 31). Tip of lobe obliquely truncated; anal plate shaped as a brown, oblong sclerite. Gonocoxite apically produced into a strong, black spine; ventromesal portion a black point (Fig. 32). Outer gonostylus club-shaped (Fig. 33). Inner gonostylus yellow; beak slender and blackened; lower beak small, nearly triangular (Fig. 34). Dorsal margin with distinct incision; dorso-lateral crest rounded. Dorsal surface medially with blackened area. Adminiculum triangular in ventral view; apex acute and split (Figs 30, 35), fused medially forming a distinct sclerite with raised base. Ventral lobe of appendage of ninth sternite blackened, roughly rounded, and provided with setae; dorsal lobe reduced into small, rounded sclerite (Fig. 35). Semen pump with central vesicle swollen (Figs 36, 37). Compressor apodeme with median incision forming a 65° angle with posterior immovable apodeme. Posterior immovable apodeme longer than compressor apodeme, basally narrow, apically flattened. Anterior immovable rounded. Intromittent organ tube-shaped, about three times as long as semen pump, basally and medially brownish-yellow, passing into yellow towards acute apex.

***Female*.** Body length 20.2–21.1 mm, wing length 16.4–17.1 mm. Generally similar to male. Antenna, if bent backward, reaching pronotum; scape, pedicel, and two basal flagellar segments yellow; remaining flagellomeres vary from yellow to bicolored. Abdomen trivittate, with broad dorsal stripe.

**Figures 38–41.**
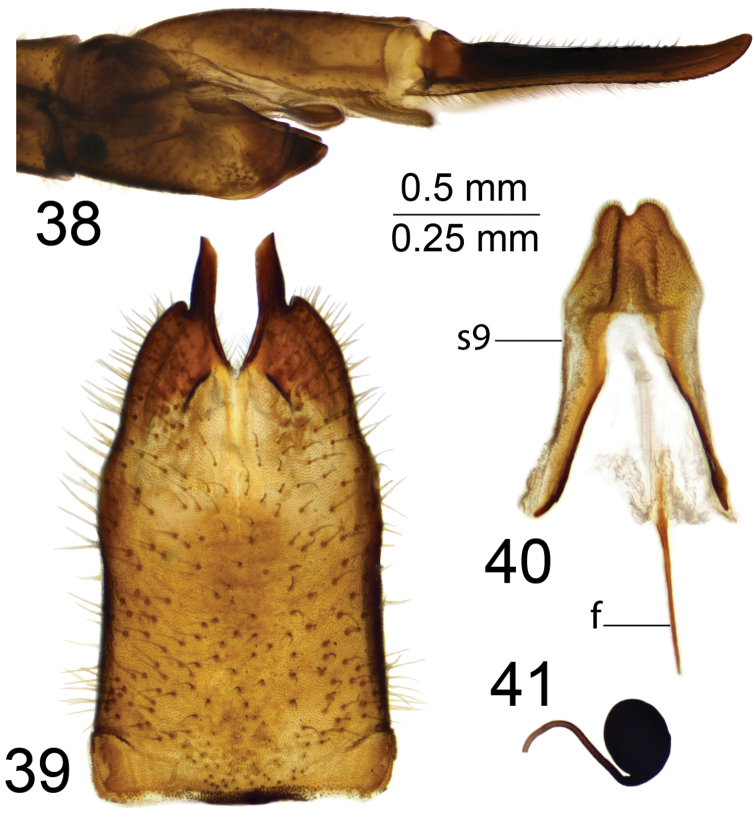
Female terminalia of T. (Vestiplex) serricauda**38** ovipositor, left lateral view **39** eighth sternite with hypovalvae, ventral view **40** ninth sternite with furca, dorsal view **41** spermatheca, dorsal view. Abbreviations: f, furca; s9, ninth sternite. Scale bars: 0.25 mm (**38**); 0.5 mm (**39–41**).

*Female terminalia*. Tenth tergite shining brown. Cercus reddish brown, nearly straight with tip narrowed and upturned; ventral margin with small, distinct serration; dorsal margin distally also with small serration (Fig. 38). Eighth sternite brown, apically darker brown (Fig. 39). Hypovalva short, dark brown, in the shape of an obliquely truncated plate. Lateral angle of eighth sternite obtuse, with small and distinct incision. Median incision between hypovalvae with serrated edge and provided with setae. Ninth sternite with lateral sclerites nearly straight, posteriorly with incision; surface covered by short setae (Fig. 40). Furca long and narrow (Fig. 40). Spermathecae nearly oval (Fig. 41).

**Figures 42–46.**
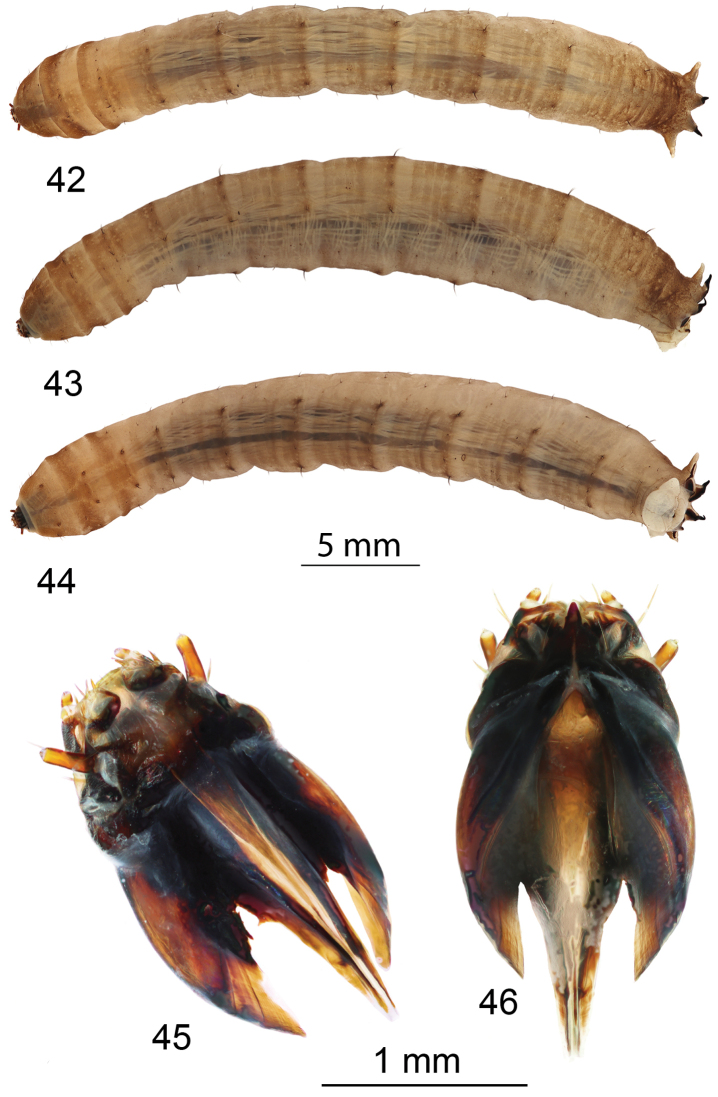
Larva of T. (Vestiplex) serricauda**42** general view, dorsal aspect **43** general view, lateral aspect **44** general view ventral aspect **45** general view of head capsule, dorsal aspect **46** general view of head capsule, ventral aspect. Scale bars: 5 mm (**42–44**); 1 mm (**45–46**).

***Larva*** (*N* = 1). Length 29 mm, width 4 mm. Body light brown (Figs 42–44).

**Figures 47–53.**
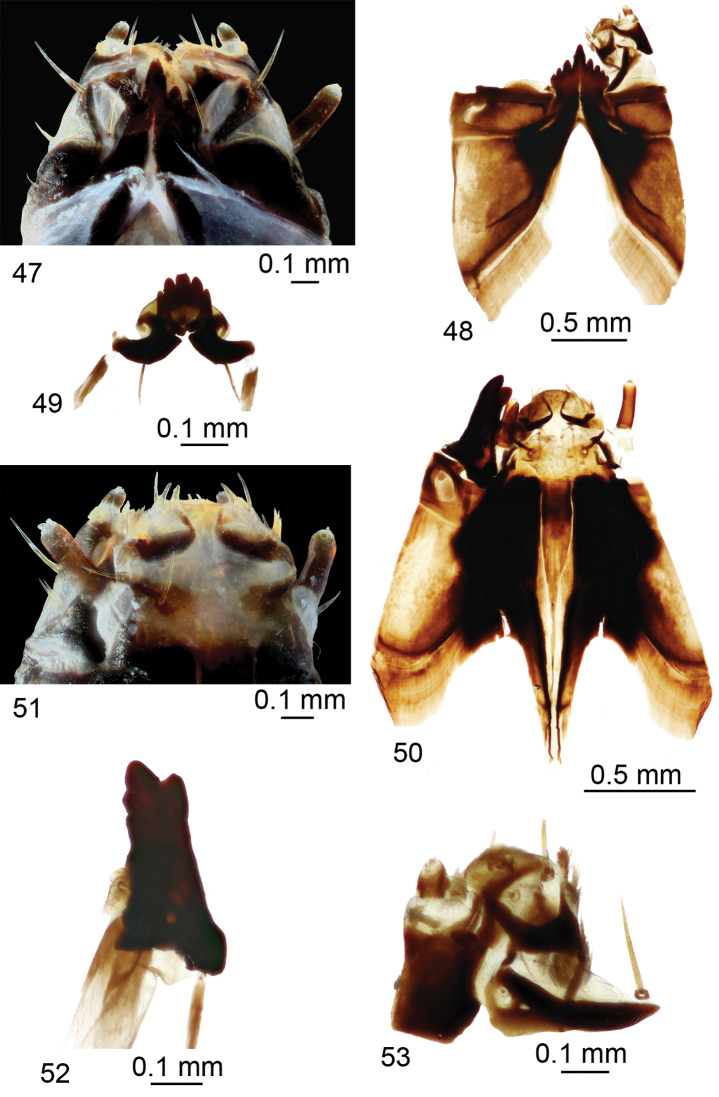
Larva of T. (Vestiplex) serricauda**47** side plates **48** hypostoma **49** hypopharynx and prementum **50** frontoclypeus **51** labrum and antennae **52** right mandible, dorsal view **53** left maxilla, ventral view. Scale bars: 0.1 mm (**47, 49, 51–53**); 0.5 mm (**48, 50**).

*Head capsule.* Length 2.3 mm, width 1.2 mm. Head capsule prognathous, hemicephalic, oval, slightly depressed dorsoventrally, and heavily sclerotized (Figs 45, 46). Internolateralia and externolateralia separated by incisions which reach almost middle of head capsule. Externolateralia widely separated ventrally (Fig. 46). Premaxillary suture separates side plate from rest of head capsule. Side plate wide, elongated, with two sensory pits and two long setae anteromedially; a short seta located posteromedially (Fig. 47). Hypostomium asymmetrical, basally fused with ventral margins of genae and side plates; bearing eight sharp teeth: four on left side, most prominent tooth in middle, and three on right side (Fig. 48). Prementum visible from below, bearing five large, sharp teeth on anterior margin; sides of prementum strongly sclerotized (Fig. 49). Labial area entirely covered with firm bristles and bearing a pair of cone-shaped palpes. Prementum dorsally fused with hemispherical and membranous hypopharynx which is covered with numerous short hairs. Lateral arm of hypopharynx curved and strongly sclerotized. Frontoclypeus fused with internolateralia. Clypeal part of frontoclypeus membranous, frontal part sclerotized. One long seta and three sensory pits on anterior part of clypeus; three short setae near inner margin of antenna (Fig. 50). Clypeolabral suture obscure. Dorsal ecdysial sutures (frontal sutures) present, meeting each other posteriorly and forming a short median coronal suture. Ecdysial sutures enclose V-shaped frontoclypeus and extend anteriorly only to base of clypeus. Labrum trapezoidal and composed of two triangular plates separated by membranous area (Fig. 50). Apical part of labrum and epipharynx covered with numerous, short hairs. Membranous part of labrum with a pair of medium-long setae in middle. Labral plates sclerotized only posteriorly and bearing numerous long firm spines on outer margin. Each plate bears a long seta, one long and two very short papillae on anterior part, one long seta almost at middle, and a sensory pit on postero-lateral part (Fig. 51). Antenna elongated, cylindrical. It has just one cylindrical segment, which is three times as long as wide at base (Fig. 51). Apically it has one small cone-shaped and several (exact number difficult to establish) small, peg-like sensillae; dorsally it has a sensory pit near middle. Mandible 1-segmented and more sclerotized than rest of head capsule, armed with four teeth (Fig. 52). Apical tooth is the most prominent; first dorsal and ventral teeth smaller than apical; second dorsal tooth smallest. Prostheca or *lacinia mobilis* present on dorsal side of mesal mandibular base; prostheca sclerotized, distinctly widening distally, and set with numerous hairs. Lateral margin of mandible with two long setae near base; a sensory pit present at base of dorsal side of mandible. Mandibles operate in horizontal plane. Conspicuous larval eye spot present below base of mandible. Maxilla consists of cardo and outer and inner lobes. Cardo wedge-shaped, bearing two long setae near distal end and a long seta near its base (Fig. 53). Outer lobe (stipes) sclerotized, except apex, which bears prominent, cylindrical palpus with several sensory structures. Short sensory structure and several long, sclerotized spines (exact number difficult to establish) on inner margin of stipes. Inner lobe (galea fused with lacinia) ventrally bears elongated, narrow sclerite extending around inner margin onto its dorsal surface; diamond-shaped sclerite present dorsally at base (Fig. 53); apical part with numerous short hairs, three long setae, and prominent sensory structure. Lacinia armed with several stout bristles and bears a ridge with sclerotized spines on outer margin.

*Thorax.* All thoracic segments wider than long. Anterior part of second and third segments covered with much denser pubescence than posterior (Figs 42–44).

*Abdomen.* First abdominal segment almost twice as long as wide. Abdominal segments II–VII almost as long as wide. All abdominal segments except last one covered by short microscopic hairs arranged into transverse rows, which are interrupted by pubescence on ventral and dorsal sides. Most macrosetae dark brown except L2 and L3. Dorsal setae D2 and D3 longest; seta D1 only slightly shorter than D2 and D3; setae D4–D6 short and appressed, more than three times shorter than D2 and D3. Setae D2 + D3 and D5 + D6 close to each other and separate from others. Lateral setae L2 and L3 very short and pale; L1 and L4 long and almost equal in length; L2 dorsolateral to L1. Setae L1, L4 more than four times as long as L2 and L3. V2 almost equal to V3 and both the longest of ventral setae. Setae V4 and V5 slightly shorter than V2 and V3. Seta V1 very short, more than five times shorter than V2 and V3.

**Figures 54–55.**
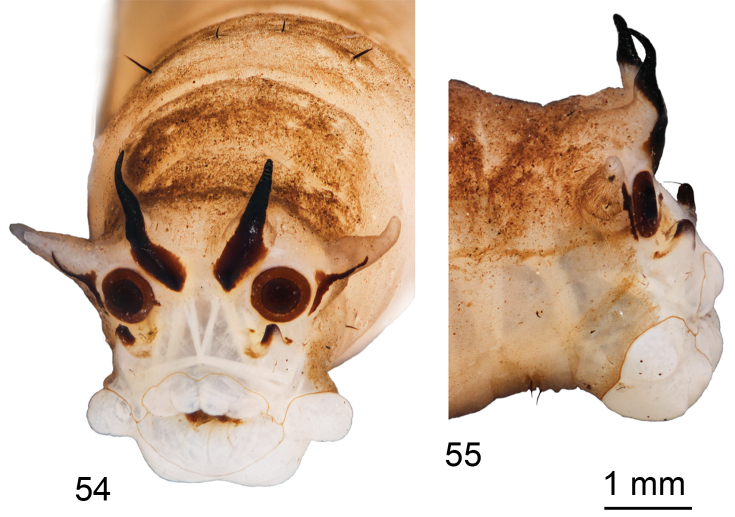
Larva of T. (Vestiplex) serricauda**54** spiracular field **55** anal division, lateral view. Scale bar: 1 mm.

*Anal division*. Dorsal and lateral lobes of spiracular disc subconical, lateral lobes in dorsolateral position (closer to dorsal than to ventral lobes). Dorsal and lateral lobes similar in length, twice as long as wide at base (Fig. 54). Ventral lobe very small, triangular, its length almost equal to width at base. Ventral lobe almost five times as short as dorsal or lateral lobe. Dorsal lobe completely sclerotized, with apex extended into acute, anteriorly directed point (Fig. 55). Lateral lobe with long, narrow, curved, dark sclerite that starts near base of spiracle and extends to mid-length of lobe; a long seta present in middle of outer edge of lobe. Ventral lobe with three small, dark spots at base; outermost spot most prominent. Distal half of lobe sclerotized, with long apical seta. Spiracle subcircular, inner circle black, outer ring brown; distance between spiracles almost twice diameter of a spiracle. Remaining area around spiracles white and glabrous. Four white, fleshy anal papillae arranged into anterior and posterior pairs. Anterior papilla broadly rounded; posterior papilla more elongated (Fig. 54). A brown band separates anus and anal papillae; this band connected to marginal band.

#### Habitat.

Larvae were found under leaf litter and woody debris accumulated on boulders. Two identical last instar larvae were collected and one of them was kept for rearing. A female emerged after 36 days and identified as T. (V.) serricauda.

#### Known distribution.

China, Japan, and South Korea (Starkevich et al. 2015; Oosterbroek 2019).

#### Remarks.

A single male belonging to T. (V.) serricauda was erroneously identified and published as T. (Mediotipula) anatoliensis Theowald, 1978 by Baek and Bae (2016) based on Korean material. The hypopygium, with broken apical part of gonocoxite (Baek and Bae 2016: fig. 2E), is identical to that of T. (V.) serricauda and can be easily recognized by the shape of inner gonostylus.

New data received from the larvae of T. (V.) serricauda once again confirm that the most important synapomorphy in the subgenus T. (Vestiplex) is a brown band separating the anus from the anal papillae, which is a unique character for this subgenus. According to the type of sclerotization of the spiracular field, larvae of the subgenus T. (Vestiplex) (based on T. (V.) nubeculosa Meigen, 1804, T. (V.) hortorum Linnaeus, 1758, T. (V.) montana Curtis, 1834, T. (V.) excisa Schummel, 1833, T. (V.) scripta Meigen, 1830, T. (V.) platymera Walker, 1856, T. (V.) arctica Curtis, 1835, T. (V.) semivittata Savchenko, 1960, and T. (V.) serricauda) can be divided into four groups. The first group includes larvae of such species as T. (V.) scripta, T. (V.) platymera, and T. (V.) semivittata. They possess sclerotized dorsal lobes with sclerotization encompassing the apices of the lobes and forming an acute, slightly anteriorly directed point. In addition, each lateral lobe has a thin, straight, and more or less vertical sclerite. The ventral lobes of this first group have sclerotized apices with several short setae and a narrow sclerotized band (with three dark spots inside) extending across the base. The second group includes T. (V.) arctica, T. (V.) nubeculosa, and T. (V.) hortorum. This group possesses sclerotized dorsal lobes but the sclerotization does not reach the apex of the lobe. Lateral and ventral lobes are very similar to that of the first group. The third group includes T. (V.) excisa and T. (V.) montana. This group of species possesses sclerotized dorsal lobes, with the sclerotization reaching the apex, but never forming anteriorly directed points. The lateral and ventral lobes are very similar to that of the first and second groups. The fourth group consists of only T. (V.) serricauda. Each dorsal lobe of this species is sclerotized both anteriorly and posteriorly, with each apex forming an acute, strongly anteriorly directed point. Each lateral lobe has a long, narrow, curved, dark sclerite, extending from near the base of a spiracle to the mid-length of each lobe. Each ventral lobe has a sclerotized distal part with a long apical seta, the base of each lobe with three small dark spots, with the narrow band missing. Sclerotization of the spiracular field of the fourth group most resembles that of larvae of T. (Triplicitipula) but not as seen in other groups of the subgenus T. (Vestiplex). The macrosetal arrangement on the dorsum and venter of the abdomen appears to be consistent in the subgenus, but the arrangement of the abdominal lateral setae is species-specific. Head capsules have never been comparatively studied in detail for the genus *Tipula* or the subgenus T. (Vestiplex); thus, comparison has been impossible among species and subgenera.

### Tipula (Vestiplex) tchukchi

Taxon classificationAnimaliaDipteraTipulidae

Alexander, 1934

F2A60671-6029-57F5-BF7D-4572F588CE78

Figs 56–64 , 65–66 , 89

Tipula (Vestiplex) tchukchi Alexander 1934: 408.
Tipula (Vestiplex) tchukchi obtusidens Savchenko 1964: 205 (synonymy after Starkevich and Paramonov 2016).
Tipula (Vestiplex) bo Mannheims 1967: 148 (synonymy after Mannheims and Savchenko 1973).

#### Type material examined.

Holotype, male, **RUSSIA**, Chukchi Peninsula, Chukotka Autonomous Okrug, Markovo township near Anadyr town, 6 July 1896, Gondatti (ZIN); paratype, female, topotypic (ZIN); paratype, male, Kamchatka Kray, mouth of Kichiga River, 27 June 1910, Skorikov (ZIN).

#### Other examined material

(Fig. 89). **NORTH KOREA**, 1 male, Seren Mts, alt. 3500 ft, 25 June 25 1938, Yankovsky (USNM); 1 female, **MONGOLIA**, Tov Aimag, Erdene Soum, Gorkhi Terelj National Park, unnamed tributary of Tuul River on its west side, 1.6 km upstream from Daichin crossing, 48.21780°N, 107.90392°E, alt. 1600 m, 9 July 2003, SRP#03070902, coll. O. Yadamsuren (ANSP).

#### Diagnosis.

Tipula (V.) thukchi can be recognized by the unarmed gonocoxite and the ninth tergite forming a concave, roughly rectangular, sclerotized saucer. The body coloration is blackish yellow, and the wing pattern is indistinct. The female has the cercus with an apical incision and outer margin rough and distinctly serrated. The eight sternite has a distinct lateral incision, and the hypovalvae are filamentous.

***Male*.** Body length 16.8 mm, wing length 17.1 mm. General body coloration blackish yellow.

*Head*. Gray, vertex and occiput gray with brown median line. Rostrum brown, dorsally dusted with gray. Nasus short. Antenna 13-segmented, if bent backward extending beyond the wing base. Scape and pedicel yellowish; first flagellar segment brownish; subsequent flagellar segments dark brown. Each flagellomere except first one with basal enlargement and moderately incised. Apical flagellomere small, reduced. Verticils shorter than corresponding segments. Palpus dark brown.

*Thorax*. Brown, dusted with grey. Pronotum blackish, gray dusted, with brown median line. Prescutum and presutural scutum brown, grey pruinose with four longitudinal stripes bordered by brown. Intermediate pair fused into brown median line. Interspace between median and lateral stripes light gray. Postsutural scutum blackish, gray pruinose with median line. Scutal lobe with two spots bordered by brown. Scutellum brown, postnotum brown, dusted with gray-brown; both sclerites with darker median line. Pleura brown, dusted with gray. Coxa brown, grey pruinose. Trochanter, femur, and tibia yellowish. Tarsal segments brown. Distal part of femur and tibia dark brown. Tarsal claws toothed. Wing pattern indistinct, only weak darkening along vein CuA. Halter yellowish, with brown knob.

*Abdomen*. Yellow. Abdominal segments 1–4 yellow, subsequent segments passing into dark brown. Tergites with lateral margins narrowly pale; dorsal stripe broad; lateral stripe pale.

**Figures 56–64.**
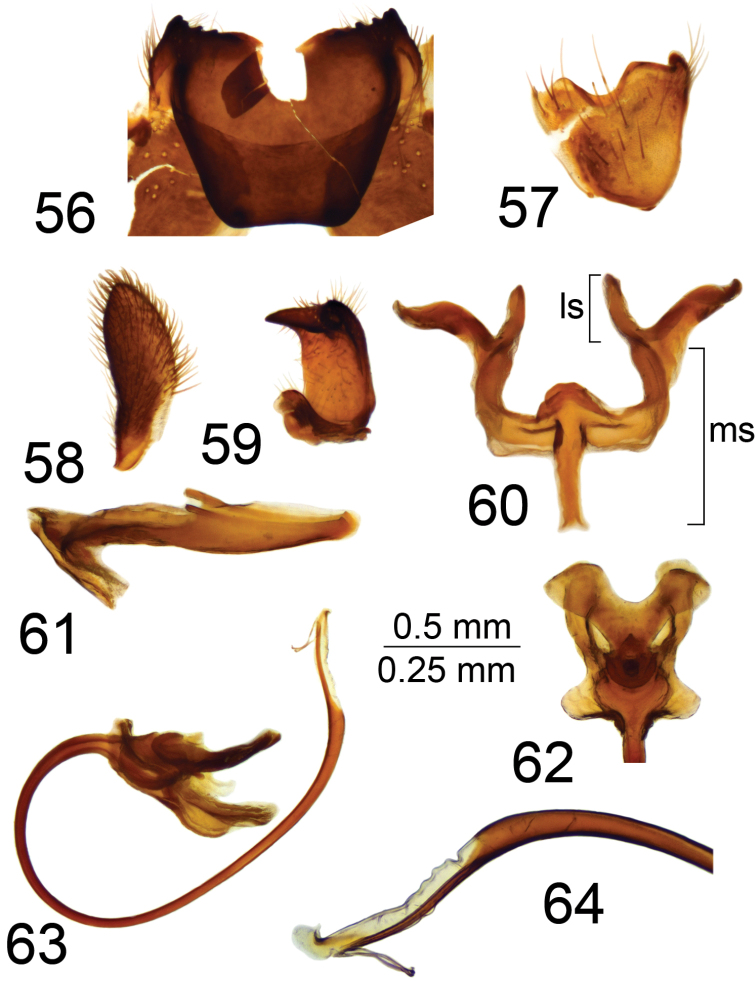
Male terminalia of T. (Vestiplex) tchukchi**56** ninth tergite, dorsal view **57** left gonocoxite, lateral view **58** left outer gonostylus **59** left inner gonostylus, lateral view **60** gonocoxal fragment, dorsal view **61** adminiculum, lateral view **62** semen pump, dorsal view **63** semen pump and intromittent organ, lateral view **64** distal part of intromittent organ, lateral view. Abbreviations: ls, lateral sclerite of gonocoxal fragment; ms, medial sclerite of gonocoxal fragment. Scale bars: 0.5 mm (**56–63**); 0.25 mm (**64**).

**Figures 65–66.**
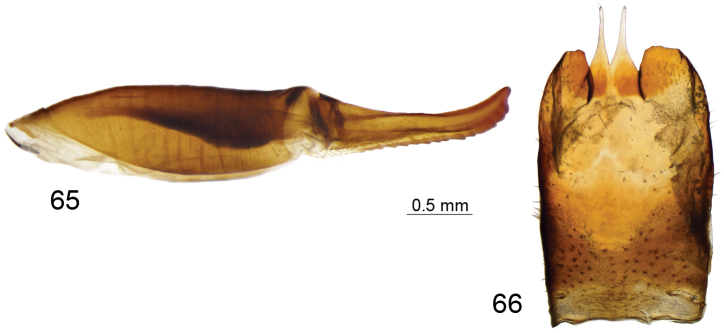
Female terminalia of T. (Vestiplex) tchukchi (Mongolia). **65** tenth tergite with cercus, left lateral view **66** eighth sternite with hypovalvae, ventral view. Scale bar: 0.5 mm.

*Hypopygium*. Brownish black. Ninth tergite forming a large, concave, roughly rectangular sclerotized saucer. Main body of tergal saucer brown and rim blackened (Fig. 56). Posterior margin of tergal saucer toothed with small denticles, broadly emarginated, with deep median U-shaped notch. Lateral angles of tergal saucer obtuse, broadly truncated. Anterior and lateral portions of tergal saucer raised into sclerotized border; border laterally produced into obtuse point directed caudad and situated under lateral angle of tergal saucer so that ninth tergite with two teeth in lateral view. Gonocoxite unarmed (Fig. 57). Outer gonostylus flattened, slightly curved, with apex rounded (Fig. 58). Inner gonostylus in the shape of a curved plate, terminating dorsally with obtuse tooth; beak claw-shaped (Fig. 59). Gonocoxal fragment large, with lateral and medial sclerites well developed (Fig. 60). Medial sclerites fused anteriorly into long, narrow apodeme; posteriorly with rounded apodeme; lateral parts broadened and arched. Lateral sclerite large and bilobed, expanded at base. Adminiculum canoe-shaped (Fig. 61). Semen pump with central vesicle relatively small and flattened (Figs 62, 63). Compressor apodeme with broad median incision, forming a 50° angle with posterior immovable apodeme. Posterior immovable apodeme approximately as long as compressor apodeme. Anterior immovable apodeme rounded. Intromittent organ tube-shaped, about four times as long as semen pump, basally and medially brown, passing into yellow towards apex. Distal part ventrally truncated, shovel-shaped, with rough edge and two stripped fragments (Fig. 65).

***Female*.** Female not known from Korean Peninsula, but can be recognized by cercus having apical incision and rough and distinctly serrated outer margin (Fig. 65). Hypovalva filamentous, flattened, broadened at base, distally pale, with short trichia at tip (Fig. 66). Median incision between hypovalvae deeper than posterior margin of eighth sternite; lateral incisions distinct.

#### Known distribution.

Finland, Sweden, Russia, and Mongolia (Oosterbroek 2019). Recorded here for the first time from the Korean Peninsula.

### Tipula (Vestiplex) verecunda

Taxon classificationAnimaliaDipteraTipulidae

Alexander, 1924

39FC66C3-0813-59F3-A879-11E1BEC43806

Figs 67–79 , 80–85 , 90

Tipula verecunda Alexander 1924: 606.
Tipula (Vestiplex) verecunda : Alexander 1935: 118; Savchenko 1964: 152; Oosterbroek and Theowald 1992: 160.

#### Type material examined.

Holotype, male, **RUSSIA**, [Sakhalin Island] Toyohara [Yuzhno-Sakhalinsk], July16, 1922, Esaki (USNM); paratypes, 3 males, 1 female, 13–14 July 1922, Esaki, topotypic (USNM); paratype, female, Shimizu [Southern Sakhalin], 27 July 1922, Esaki (USNM); paratype, female, Odasam [Southern Sakhalin], 31 July 1922, Esaki (USNM).

#### Other examined material

(Fig. 90). **SOUTH KOREA**, 5 males, 3 females, Jeju-do, Jeju Island, Seogwipo, Saekdal-dong, 33°21.45'N, 126°27.85'E, alt. 1100 m, 18 June 2019, H.-Y. Seo (NIBR); 6 males, Seogwipo, Saekdal-dong, 35°21.62'N, 126°27.76'E, alt. 1100 m, 18 June 2019, S. Podenas (NIBR). **CHINA**, 1 male, Shaanxi, Qinling Mts, Hauzherza vill., 33°52.42'N, 107°48.77'E, alt. 1600 m, 2–3 June 2009, leg. Saldaitis & Floriani (NRC).

#### Diagnosis.

Tipula (V.) verecunda can be easily recognized by the eighth sternite laterally having long setae and the ninth sternite being ventrally produced into a small tubercle. The ninth tergite has a U-shaped notch posteriorly, and anterior to the notch, the tergite is divided by a pale membrane. The ventral portion of the ninth tergite is yellow, with a pair of blackened narrow plates, and the gonocoxite is unarmed. The thorax is grey, with four darker grey stripes narrowly bordered by brown, and the wing is distinctly patterned with dark brown. The female can be recognized by the cercus having a smooth margin and a long, blade-shaped hypovalvae.

***Male*.** Body length 17.4–21.6 mm, wing length 20.0–24.5 mm. General body coloration brownish yellow.

*Head*. Vertex and occiput ochraceous yellow, with dark median line. Rostrum dark brown, dorsally narrowly ochraceous yellow. Nasus distinct. Antenna 13-segmented, if bent backward extending beyond the wing base. Scape and pedicel yellow; flagellum brownish black. Each flagellomere, except for first one, with weak basal enlargement. Apical flagellomere small, reduced. Verticils longer than corresponding segments. Palpus dark brown.

*Thorax*. Pronotum ochraceous yellow, with dark median spot. Prescutum and presutural scutum grey, with four darker grey stripes, narrowly bordered by brown. Intermediate pair fused anteriorly. Interspace ochraceous light yellow. Postsutural scutal lobe with two ochraceous brownish yellow spots bordered by brown. Scutellum brown, with broad, dark brown median line. Postnotum brownish, with pale median line, and dusted with gray. Pleura yellowish. Wing distinctly patterned with dark brown. Halter pale, with brownish-black knob. Coxa yellowish; trochanter yellow; femur basally yellow, passing into brown towards dark brown tip. Tibia and tarsal segments brownish black. Tarsal claw with tooth.

*Abdomen*. Abdominal segments 1–4 yellow; segment 5 blackish, yellowish laterally; remaining segments black. Dorsal stripe on first tergite broad, on tergites 2–4 pale, on fifth tergite black. Lateral abdominal stripe distinct.

**Figures 67–79.**
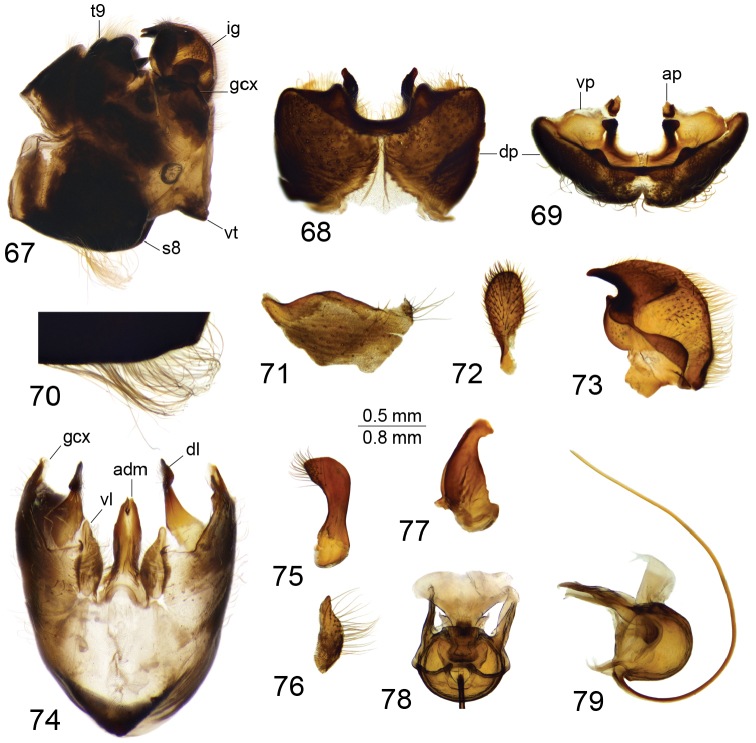
Male terminalia of T. (Vestiplex) verecunda**67** hypopygium, lateral view **68** ninth tergite, dorsal view **69** ninth tergite, caudal view **70** eighth sternite, lateral view **71** left gonocoxite, lateral view **72** left outer gonostylus **73** left inner gonostylus, lateral view **74** ninth sternite, ventral view (ninth tergite, outer and inner gonostyles removed) **75** right dorsal lobe of appendage of ninth sternite **76** right ventral lobe of appendage of ninth sternite **77** adminiculum, lateral view **78** semen pump, dorsal view **79** semen pump and intromittent organ, lateral view. Abbreviations: adm, adminiculum; ap, anal plate; dl, dorsal lobe of appendage of ninth sternite; dp, dorsal portion of ninth tergite; gcx, gonocoxite; gcx, gonocoxite; ig, inner gonostylus; og, outer gonostylus; s8, eighth sternite; vl, ventral lobe of appendage of ninth sternite; vp, ventral portion of ninth tergite; vt, ventral tubercle of ninth sternite. Scale bars: 0.8 mm (**67**); 0.5 mm (**68–79**).

*Hypopygium*. Black (Fig. 67). Ninth tergite posteriorly with deep U-shaped notch, anteriorly to notch divided by pale membrane, dorsal portion dark brown, posterior margin provided with setae, additional short projection on either side of midline (Figs 68, 69). Ventral portion yellow with a pair of blackened and microscopically serrated narrow plates; tip of plate pointed outward. Anal plate shaped as a small brown sclerite (Fig. 68). Gonocoxite unarmed, irregular in outline (Fig. 71). Outer gonostylus nearly oval, with basal part narrowed (Fig. 72). Inner gonostylus in the shape of rounded sclerite, terminating into a short upper beak with a small lower beak; beaks separated by round incision (Fig. 73). Dorsal crest with yellow setae and short, black spines grouped on dorsal surface, edge basally bent outward. Adminiculum nearly parallel-sided in ventral view, fused medially forming a distinct sclerite (Fig. 74). Basal part of adminiculum broadened and raised; apex funnel-shaped, with preapical incision in ventro-lateral view (Fig. 77). Ninth sternite ventrally produced into small tubercle (Figs 67, 74). Appendage of ninth sternite with ventral lobe oblong, tip narrowed, surface with setae (Figs 74, 76). Dorsal lobe in the shape of flattened, curved plate; tip on inner surface provided with setae (Figs 74, 75). Eighth sternite posteriorly with pale median area; laterally provided with long setae reaching 1.1 mm long. Semen pump with central vesicle swollen (Figs 78, 79). Compressor apodeme flattened forming a 50° angle with posterior immovable apodeme. Posterior immovable apodeme shorter than compressor apodeme, narrowed. Anterior immovable apodeme narrow. Intromittent organ tube-shaped, about three times as long as semen pump, basally and medially brownish yellow, passing into yellow towards acute apex.

***Female*.** Body length 27.3–28.7 mm, wing length 23.0–24.5 mm. Generally similar to male. Thorax brown. Abdomen with distinct median stripe. Tergites and sternites with lateral margin pale.

**Figures 80–85.**
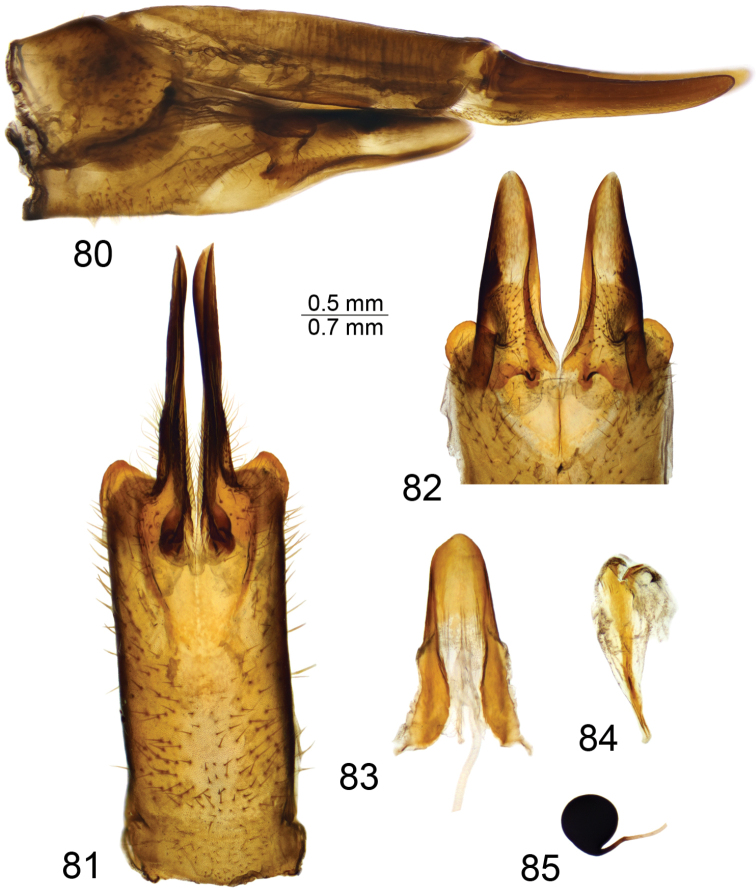
Female terminalia of T. (Vestiplex) verecunda**80** ovipositor, left lateral view **81** eighth sternite with hypovalvae, ventral view **82** distal part of eighth sternite with hypovalvae, ventral view **83** ninth sternite, dorsal view **84** furca, dorsal view **85** spermatheca, dorsal view. Scale bars: 0.7 mm (**80, 82**); 0.5 mm (**81, 83–85**).

*Female terminalia*. Tenth tergite basally shining brown but on other two-thirds shining black. Cercus reddish brown, nearly straight, with tip narrowed; ventral margin smooth (Fig. 80). Eighth sternite with hypovalva long, blade-shaped, basally with setae (Figs 80–82). Lateral angle of eighth sternite flattened and slightly extended. Ninth sternite with posterior half funnel-shaped, rounded apically (Fig. 83). Furca in the shape of a pale stripe narrowed anteriorly (Fig. 84). Spermatheca spherical (Fig. 85).

#### Known distribution.

Russia, Japan, and China (Oosterbroek 2019). Recorded here for the first time from South Korea.

**Figures 86–90.**
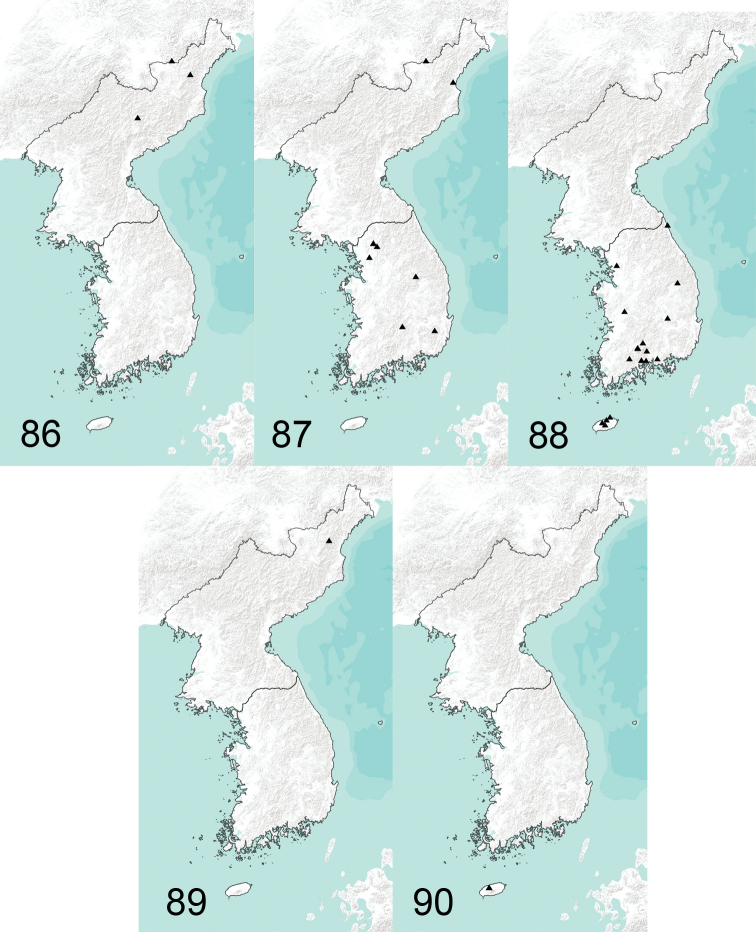
Collecting sites of T. (Vestiplex) in Korean Peninsula **86**T. (V.) coquillettiana**87**T. (V.) kuwayamai**88**T. (V.) serricauda**89**T. (V.) tchukchi**90**T. (V.) verecunda.

## Supplementary Material

XML Treatment for Tipula (Vestiplex)

XML Treatment for Tipula (Vestiplex) coquillettiana

XML Treatment for Tipula (Vestiplex) kuwayamai

XML Treatment for Tipula (Vestiplex) serricauda

XML Treatment for Tipula (Vestiplex) tchukchi

XML Treatment for Tipula (Vestiplex) verecunda
